# Evaluation of Various Diagnostic Strategies for Bacterial Vaginosis, Including a New Approach Based on MALDI-TOF Mass Spectrometry

**DOI:** 10.3390/microorganisms12010111

**Published:** 2024-01-05

**Authors:** Linda Abou Chacra, Hortense Drouet, Claudia Ly, Florence Bretelle, Florence Fenollar

**Affiliations:** 1 Campus Santé Timone, Aix-Marseille University, IRD, AP-HM, SSA, VITROME, 13005 Marseille, France; abouchacra.linda@gmail.com (L.A.C.); hortensedrouet@gmail.com (H.D.); claudiaks_ly@hotmail.com (C.L.); 2 IHU-Méditerranée Infection, 13005 Marseille, France; 3 Campus Santé Timone, Aix-Marseille University, IRD, AP-HM, MEPHI, 13005 Marseille, France; florence.bretelle@ap-hm.fr; 4 Department of Gynaecology and Obstetrics, Gynépole, La Conception, AP-HM, 13005 Marseille, France

**Keywords:** bacterial vaginosis, dysbiosis, vaginal discharge, Amsel criteria, Nugent score, qPCR *Fannyhessea vaginae*, qPCR *Gardnerella vaginalis*, molecular biology, MALDI-TOF, mass spectrometry

## Abstract

Bacterial vaginosis (BV) is a common dysbiosis of unclear etiology but with potential consequences representing a public health problem. The diagnostic strategies vary widely. The Amsel criteria and Nugent score have obvious limitations, while molecular biology techniques are expensive and not yet widespread. We set out to evaluate different diagnostic strategies from vaginal samples using (1) a combination of abnormal vaginal discharge and vaginal pH > 4.5; (2) the Amsel-like criteria (replacing the “whiff test” with “malodorous discharge”); (3) the Nugent score; (4) the molecular quantification of *Fannyhessea vaginae* and *Gardnerella vaginalis* (qPCR); (5) and MALDI-TOF mass spectrometry (we also refer to it as “VAGI-TOF”). Overall, 54/129 patients (42%) were diagnosed with BV using the combination of vaginal discharge and pH, 46/118 (39%) using the Amsel-like criteria, 31/130 (24%) using qPCR, 32/130 (25%) using “VAGI-TOF”, and 23/84 (27%) using the Nugent score (not including the 26 (31%) with intermediate flora). Of the 84 women for whom the five diagnostic strategies were performed, the diagnosis of BV was considered for 38% using the combination of vaginal discharge and pH, 34.5% using the Amsel-like criteria, 27% using the Nugent score, 25% using qPCR, and 25% using “VAGI-TOF”. When qPCR was considered as the reference, the sensitivity rate for BV was 76.2% for the combination of vaginal discharge and pH, 90.5% for the Amsel-like criteria, 95.2% for the Nugent score, and 90.5% for “VAGI-TOF”, while the specificity rates were 74.6%, 84.1%, 95.3%, and 95.3%, respectively. When the Nugent score was considered as the reference, the sensitivity for BV was 69.6% for the combination of vaginal discharge and pH, 82.6% for the Amsel-like criteria, 87% for qPCR, and 78.7% for “VAGI-TOF”, while the specificity rates were 80%, 94.3%, 100%, and 97.1%, respectively. Overall, the use of qPCR and “VAGI-TOF” provided a consistent diagnosis of BV, followed by the Nugent score. If qPCR seems tedious and for some costly, “VAGI-TOF” could be an inexpensive, practical, and less time-consuming alternative.

## 1. Introduction

Bacterial vaginosis is a common cause of vaginal discharge [1]. Its prevalence rates range from 10% to 30% of women [2] and up to 50% among women who have sex with women [3]. Bacterial vaginosis is considered a particular reversal of the female vaginal microbiome via the suppression of *lactobacilli* and an overgrowth of resident facultative and strict anaerobic bacteria such as *Gardnerella vaginalis*, *Fannyhessea vaginae*, *Mobiluncus curtisii*, *Prevotella bivia*, and *Megasphaera* type I [4,5].

There has been little success in terms of identifying the origin of this dysbiosis, although some potential predisposing characteristics have been recognised, such as the use of copper intrauterine devices [6], sexual behaviour [7], and personal hygienic behaviour, including douching [8]. This dysbiosis is occasionally asymptomatic [9,10,11]. In a national health and nutrition survey conducted in the United States between 2001 and 2004, 21 million (29.2%) women were diagnosed with bacterial vaginosis, 84% of whom reported no symptoms [12].

Untreated bacterial vaginosis can lead to serious obstetric and gynaecological complications such as miscarriage, chorioamnionitis, and premature birth [13,14], as well as an increased risk of contracting sexually transmitted pathogens such as human immunodeficiency virus and human papillomavirus [15,16]. Thus, bacterial vaginosis must be properly diagnosed and treated. The Amsel criteria [17] and the Nugent score [18] are classically used by physicians. Both diagnostic tools have obvious limitations, such as the need for good technical skills to prepare good quality smears and to analyze the results accurately for the Nugent score. Although new molecular biology tools were introduced several years ago, their use remains limited and expensive [19,20,21,22,23,24,25,26,27,28].

However, diagnostic problems remain dominant in clinical practice and present a significant challenge. In this context, matrix-assisted laser desorption ionization–time of flight mass spectrometry (MALDI-TOF MS) is a technology that has emerged in recent years to identify microorganisms by analyzing their protein profiles [29]. This tool allows for the rapid, reproducible, and low-cost identification of isolated strain samples such as blood cultures [30] and urine [31]. Therefore, it appeared interesting to evaluate this tool for use on vaginal samples to determine whether it is possible to obtain typical protein profiles of normal flora and bacterial vaginosis.

Our main objective was to evaluate multiple strategies for the diagnosis of bacterial vaginosis, from a combination of abnormal vaginal discharge and vaginal pH to a new strategy based on MALDI-TOF mass spectrometry to improve women’s medical care.

## 2. Materials and Methods

### 2.1. Sample Collection and Ethical Approval

All vaginal samples were collected using a Sigma Transwab^®^ swab (Medical Wire, Corsham, UK) during gynaecological consultations and sent for diagnosis to our clinical microbiology laboratory (University hospitals of Marseille, AP-HM, Marseille, France). Patients were informed of the possible use of their samples and data collected during their care for research purposes, as permitted under French law (article L.1211-2 of the French Public Health Code). They were given the opportunity to object by notifying the DPO of the AP-HM. All data used were rendered anonymous. Our independent ethics committee (IEC No. 09-007 and IEC No. 2022-034) approved the clearance of the Ethics Review Committee (ERC).

### 2.2. Diagnostic Tools

Five diagnostic strategies were compared.

#### 2.2.1. Combination of Vaginal Discharge and Vaginal pH

When the vaginal pH, measured using an EcoCare^®^ pH-cotton stick (Merete Medical, Berlin, Germany), was greater than 4.5 and was associated with the presence of abnormal vaginal discharge, a diagnosis of bacterial vaginosis was considered.

#### 2.2.2. Amsel-Like Criteria

Amsel’s criteria are a combination of four clinical criteria [17]: (1) greyish-white homogeneous vaginal discharge; (2) a pH of vaginal secretions > 4.5; (3) the release of an amine-like odor (fishy smell) after adding a drop of 10% KOH (potassium hydroxide) solution to a drop of vaginal secretion (“whiff test”); (4) the presence of clue cells (vaginal squamous epithelial cells coated with Gram-variable Coccobacilli). As the “whiff test” is rarely, if ever, used in clinical practice in our hospital, the Amsel-like criteria were simplified by replacing the “whiff test” with the presence or absence of malodorous vaginal discharge. If the patient met three of the four criteria, she was classified as having bacterial vaginosis.

#### 2.2.3. Nugent Score

The Gram staining was performed with the Color Gram-2 Kit (bioMérieux, Marcy-l’Etoile, France). To summarize, four steps were performed: (1) staining with Gentian violet for one minute before rinsing; (2) etching with Lugol for one minute before rinsing; (3) rapid flooding for ten seconds with a bleaching agent and alcohol before rinsing; (4) counterstaining with Fuchsine for one minute before rinsing. The prepared slides were observed through an optical microscope (Leica^®^ DM 1000; Leica Microsystems, Wetzlar, Germany) with a 100× magnification objective. The samples were classified based on the Nugent score [18], with 0–3 considered normal flora, 4–6 as intermediate flora, and 7–10 as bacterial vaginosis.

#### 2.2.4. Quantitative Real-Time Polymerase Chain Reaction (qPCR)

Molecular biology diagnoses of bacterial vaginosis were based on a real-time quantitative PCR assay determining DNA levels of *Fannyhessea vaginae* and *Gardnerella vaginalis* [27,28]. This technique has been used in our routine diagnoses of bacterial vaginosis since 2009. It was considered in this study as the reference tool for the diagnosis of bacterial vaginosis.

##### DNA Extraction

The DNA extraction was performed on an EZ1 automate (Qiagen, Courtaboeuf, France) using a commercial extraction kit, the QIAamp Tissue Kit^®^ (Qiagen), following the manufacturer’s instructions. The DNA was extracted from 200 µL of vaginal sample digested with 200 μL of G2 buffer and 10 μL of proteinase K at 56 °C for 20 min, then eluted in 100 µL of distilled water.

##### Quantitative PCR Assay

Each reaction mixture contained 15 µL of PCR mix (10 μL of Eurogentec™ Probe PCR Master Mix (Eurogentec, Liege, Belgium); 3 μL of DNAse- and RNAse-free distilled water; and 0.5 μL of each reverse and forward primer (50 μM), probe (50 μM), and Uracil DNA glycosylase (UDG)) and 5 µL of extracted DNA.

A quantitative PCR assay was carried out with a CFX96 real-time system (Bio-Rad Laboratories, Foster City, CA, USA). We used the following amplification program: incubation at 50 °C for two minutes (for UDG activation) and initial DNA denaturation at 95 °C for five minutes, followed by a series of 39 cycles consisting of DNA denaturation at 95 °C for five seconds and primer annealing probe hybridization at 60 °C for 30 s.

To validate the qPCR run, we used circular plasmid DNA samples (named 393, which is a plasmid pUC57 of 2710 bp in which a target sequence of 393 bp is cloned) as the positive controls and master mixtures as the negative controls for each assay.

According to the methodology presented by Menard et al. [27], the samples were defined as having bacterial vaginosis if the level of *F. vaginae* DNA was ≥10^8^ copies/mL or the level of *G. vaginalis* DNA was ≥10^9^ copies/mL.

#### 2.2.5. MALDI-TOF Mass Spectrometry (“VAGI-TOF”)

One microliter of each vaginal sample was deposited onto a MALDI-TOF target (plate containing 96 spots) in six spots without any pretreatment, homogeneously and forming a thin layer to obtain a good acquisition. Once the vaginal deposit had dried, it was covered with 1.5 μL of the matrix solution to co-crystallize with the sample. The matrix consisted of four reagents: (1) α-cyano-4-hydroxycinnamic saturated acid or HCCA (Sigma, Lyon, France); (2) 50% HPLC-grade acetonitrile (VWR, Strasbourg, France); (3) 25% trifluoroacetic acid or TFA (Aldrich, Dorset, UK); (4) 25% HPLC-grade water (VWR, Strasbourg, France).

The analysis was performed on a Microflex LT^®^ MALDI-TOF mass spectrometry device (Bruker Daltonics, Bremen, Germany) and its automatic module, which made it possible using the FlexControl acquisition software v. 3.4 (Build 135) and MALDI Biotyper Compass analysis software v. 4.1 (Version (80) to acquire protein spectra.

To establish a comprehensive understanding of the sample under investigation, an average reference peak list designated as the main spectrum (MSP) was calculated for each on the basis of the six MALDI-TOF spectra acquired on it with MBT Compass Explorer software version 4.1, build 80 (Bruker, Bremen, Germany), using the BioTyper MSP Creation Standard Method v1.2.

Thus, 38 MSP were calculated and selected in order to calculate a dendrogram using the Biotyper MSP Dendrogram Creation Standard Method v1.4, with the following parameters: measure of distance by correlation and average linkage. The calculated dendrogram presents the relative similarity of each MSP between each other by assigning distances of clusterization based on the matching of peak patterns between spectra.

### 2.3. Statistical Methods

Descriptive statistics (frequency, percentage) were used for the categorical variable. The mean and standard deviation were calculated for continuous variables. Fisher’s exact tests were used to compare frequencies. All analyses were based on two-sided *p* values, with statistical significance defined by *p* ≤ 0.05. Statistical analyses were performed with biostaTGV statistical software (accessed on 01/11/2023). Sensitivity, specificity, and likelihood ratios for diagnostic performance using categorical data were performed using MedCalc (http://www.medcalc.org/calc/diagnostic_test.php; accessed on 01/11/2023).

## 3. Results

### 3.1. Main Patient Characteristics

Of the 130 vaginal samples from the 130 women analyzed, 110 were from women of childbearing age with no current pregnancies, 17 from pregnant women, and three from postmenopausal women. The main clinical characteristics of the patients are summarized in Table 1. The median age was 28 years (range of 18–54 years old). A previous history of bacterial vaginosis was reported in 34 (26%) women. Abnormal vaginal discharges were the main manifestation, accounting for 47.7% (62/130).

### 3.2. Diagnosis of Bacterial Vaginosis According to the Different Strategies

**Figure 1:**
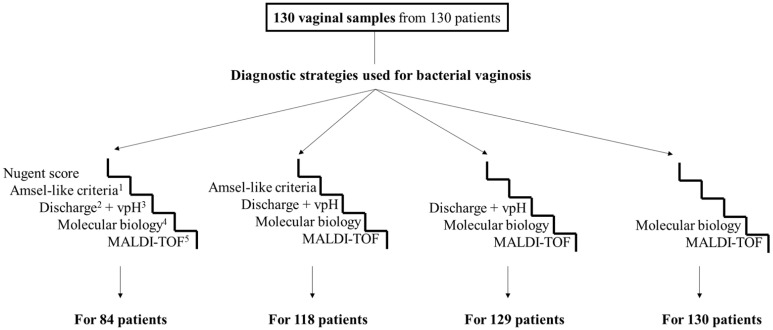
Flow chart of the different diagnostic strategies used for bacterial vaginosis and the number of samples analyzed by each. ^1^ Amsel-like criteria (replacement of the “whiff test” by the presence of a malodorous vaginal discharge). ^2^ Abnormal vaginal discharge. ^3^ Vaginal pH. ^4^ Quantitative real-time PCR targeting *F. vaginae* and *G. vaginalis*. ^5^ Matrix-assisted laser desorption ionization–time of flight (MALDI-TOF) mass spectrometry.

**Figure 2:**
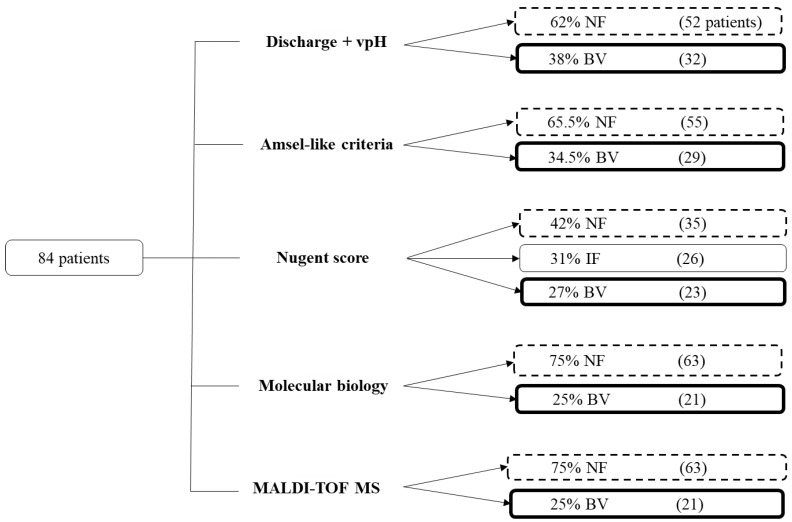
Overall results for the 84 patients who benefited from the five strategies for diagnosing bacterial vaginosis. NF: normal flora; IF: intermediate flora; BV: bacterial vaginosis.

Of the 130 vaginal samples from 130 patients analyzed via qPCR and MALDI-TOF mass spectrometry, 129 were also analyzed using a combination of abnormal vaginal discharge and vaginal pH > 4.5, 118 using Amsel-like criteria, and 84 using the Nugent score (Figure 1 and Figure 2). Furthermore, using molecular biology, it was shown that 31 out of 130 patients (24%) suffered from bacterial vaginosis (eight of them (25.8%) had no abnormal vaginal discharge; Supplementary Table S1), while according to the MALDI-TOF mass spectrometry results, 32 out of the 130 patients (25%) suffered from bacterial vaginosis. Using the Nugent scores, 23 out of 84 patients (27%) were diagnosed with bacterial vaginosis and 26 (31%) had intermediate vaginal flora. Using the Amsel-like criteria, 46 out of 118 patients (39%) were diagnosed with bacterial vaginosis. Finally, using a combination of vaginal discharge and vaginal pH > 4.5, 54 out of 129 patients (42%) were diagnosed with bacterial vaginosis.

### 3.3. Performance of Diagnostic Strategies for Bacterial Vaginosis

Of the 84 women for whom the four diagnostic strategies detailed below were carried out, diagnoses of bacterial vaginosis were considered for 38% (32/84) using the combination of vaginal discharge and vaginal pH, 34.5% (29/84) using the Amsel-like criteria, 27% (23/84) using the Nugent score, and 25% (21/84) using the qPCR assay (Figure 2). The concordance rate between these four diagnostic strategies in terms of the presence or absence of bacterial vaginosis was 50% (42/84) (Table 2). The four diagnostic tools (excluding “VAGI-TOF”) were first compared, with the Nugent score serving as the reference diagnostic tool, followed by molecular biology (Table 3).

If we take the Nugent score as the reference diagnostic tool for bacterial vaginosis then (1) the combination of vaginal discharge and vaginal pH would have a sensitivity rate of 69.6%, a specificity rate of 80%, a positive predictive value of 69.6%, and a negative predictive value of 80%; (2) the Amsel-like criteria would have a sensitivity rate of 82.6%, a specificity rate of 94.3%, a positive predictive value of 90.5%, and a negative predictive value of 89.2%;(3) and the molecular biology assay would have a sensitivity rate of 87%, a specificity rate of 100%, a positive predictive value of 100%, and a negative predictive value of 92.1%.

If we take the molecular biology assay as the reference diagnostic tool for bacterial vaginosis then (1) the combination of vaginal discharge and vaginal pH would have a sensitivity rate of 76.2%, a specificity rate of 74.6%, a positive predictive value of 50% and a negative predictive value of 90.4%; (2) the Amsel-like criteria would have a sensitivity rate of 90.5%, a specificity rate of 84.1%, a positive predictive value of 65.5%, and a negative predictive value of 96.4%; (3) and the Nugent score would have a sensitivity rate of 95.2%, a specificity rate of 95.2, a positive predictive value of 87%, and a negative predictive value of 98%.

### 3.4. Development of a New Diagnostic Tool Using Mass Spectrometry, “VAGI-TOF”

#### Reproducibility and Global Aspect Ratio of the Spectra

**Figure 3:**
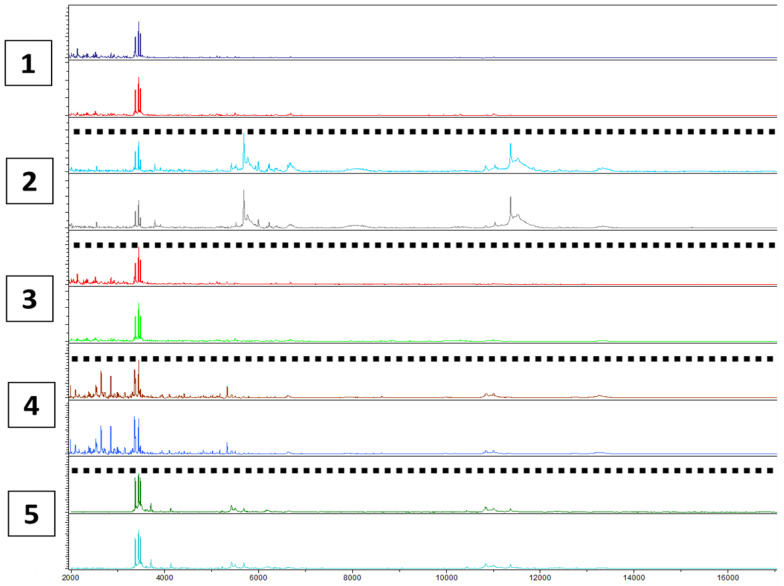
Reproducibility of MALDI-TOF mass spectrometry by comparing the spectra of five different vaginal swabs before and after freezing at −80 °C.

In the spectra preprocessing stage, 130 peaks were analyzed using the binary discriminant method to identify their discriminating power and threshold. Each vaginal sample was also tested directly and then after storage at −80 °C for two months without the addition of any protective agent. This process was conducted to evaluate the reproducibility of this technique and the ability to work on previously frozen samples (Figure 3).

**Figure 4:**
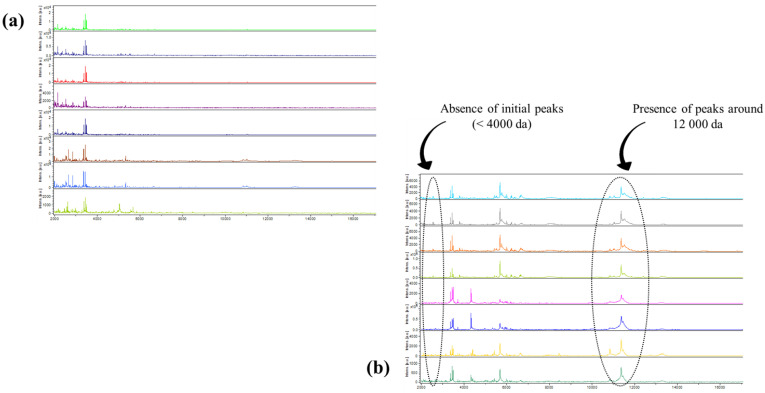
Comparison of the general appearances of the spectra of women with normal vaginal flora (**a**) and those with bacterial vaginosis (**b**) using MALDI-TOF mass spectrometry as a diagnostic tool.

**Figure 5:**
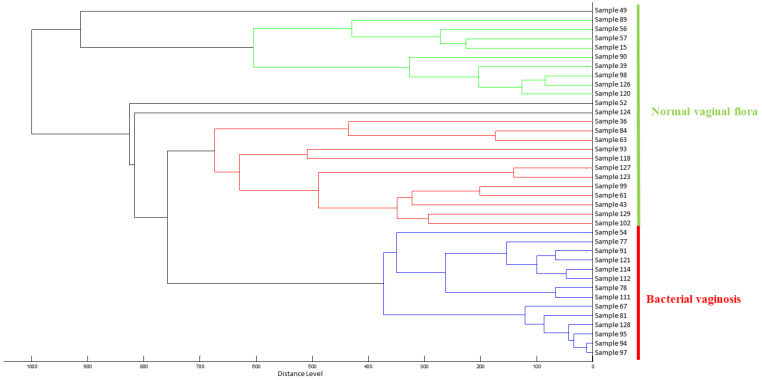
MALDI-TOF mass spectrometry dendrogram of 14 bacterial vaginosis and 24 normal vaginal flora confirmed using three diagnostic methods (Amsel-like criteria, Nugent score, and molecular biology).

From the very first analyses, our attention was drawn to the fact that all spectra from the women with a diagnosis of bacterial vaginosis (confirmed by the molecular method) had similar appearances. Therefore, the classification criteria for samples identifying bacterial vaginosis were predominantly based on a consistent initial absence of peaks (<4000 da) and a frequent presence of peaks around 12,000 da, as depicted in Figure 4. A dendrogram based on the dichotomization of the 130 peaks was then generated. The mass spectrometry data output showed the clustering of each group for normal flora versus bacterial vaginosis. Subsequently, 32/130 (25%) had bacterial vaginosis (Figure 5).

### 3.5. Analysis of the Spectra According to the Diagnostic Tools Already Used

This new tool was compared with the four others previously applied to the diagnosis of bacterial vaginosis. If we took the combination of vaginal discharge and vaginal pH as the reference diagnostic tool, “VAGI-TOF” would have a sensitivity of 46.3%, a specificity of 90.7%, a positive predictive value of 78.1%, and a negative predictive value of 70.1%. If we took Amsel-like criteria as the reference diagnostic tool, “VAGI-TOF” would have a sensitivity of 54.3%, a specificity of 95.8%, a positive predictive value of 89.3%, and a negative predictive value of 76.7%. If we took the Nugent score as the reference diagnostic tool, “VAGI-TOF” would have a sensitivity of 78.7%, a specificity of 97.1%, a positive predictive value of 94.7% and a negative predictive value of 87.2%. If we took the molecular biology as the reference diagnostic tool, “VAGI-TOF” would have a sensitivity of 93.6%, a specificity of 97%, a positive predictive value of 90.6% and a negative predictive value of 98% (Table 4).

## 4. Discussion

The complex polymicrobial nature, the presence of troublesome symptoms such as abnormal vaginal discharge, and the possible absence of symptoms associated with an imbalance of the vaginal microbiome in patients with bacterial vaginosis make its diagnosis difficult [32,33]. Its diagnosis and management are, therefore, difficult for physicians. A wide variety of diagnostic strategies have been reported to be used by physicians [27,34]. However, the potential risks associated with bacterial vaginosis and the difficulties in managing them make it important to have reliable diagnostic tools at our disposal. Even within our laboratory, different diagnostic strategies are carried out according to the specific requests of physicians (Gram staining, the Nugent score, or molecular biology). Molecular biology, based on the quantitative PCR analysis of *F. vaginae* and *G. vaginalis*, has become an important tool in our laboratory for diagnosing bacterial vaginosis. This is partly because we developed it and partly because this rational, reproducible technique is precise and free from human error. However, not all physicians prescribe it, either because they are unfamiliar with it, they find it expensive, they prefer the Nugent score data, or they rely on a vaginal pH > 4.5 and the presence of abnormal vaginal discharge to establish the diagnosis of bacterial vaginosis.

In this study, we compared the efficacy of five diagnostic strategies of bacterial vaginosis: (1) one that could be carried out by the physician seeing the patient in consultation; (2) one requiring a combination of clinical and microscopic intervention; (3) one to be carried out in the laboratory by specially trained technicians or microbiologists; (4) one to be carried out in a molecular biology laboratory; (5) one to be carried out in a laboratory performing MALDI-TOF mass spectrometry. While the first diagnostic techniques have already been carried out with slight differences, the latest technique (MALDI-TOF mass spectrometry) has never been applied.

Firstly, it was observed that the pH level increases in patients with bacterial vaginosis [35,36] and patients showing abnormal vaginal discharges [37]. One question was whether vaginal pH measurements, which are associated with the presence of abnormal vaginal discharges, could be used as a simple diagnostic strategy for bacterial vaginosis. Indeed, during the consultation, and after questioning the patient, the vaginal pH could be measured, enabling the diagnosis to be made directly and treatment to be proposed to the patient without waiting for laboratory results. In current clinical practice, one of the four Amsel criteria, the “whiff” test, is rarely, if ever, performed, at least in our area. We, therefore, decided to replace this criterion with the search for the presence of malodorous vaginal discharge during the consultation as an alternative. It should be noted that the Amsel criteria also require a direct examination of the vaginal swab for clue cells. In any case, according to our results, the combination of abnormal vaginal discharge and vaginal pH, as well as the Amsel-like criteria, had low specificity and also low sensitivity. A significant number of patients with bacterial vaginosis and laboratory dysbiosis have no clinical manifestations, while the presence of abnormal vaginal discharge and increased vaginal pH is not synonymous with bacterial vaginosis [9,38]. Both diagnostic strategies are ineffective and unreliable.

Although the Nugent score has a comparatively high specificity rate (93%), some of the vaginal microbiome observed in a sample will not be classified as either bacterial vaginosis or normal vaginal flora but as intermediate flora. There is currently real ambiguity as to the true classification of this so-called “intermediate” flora. Some consider it to be bacterial vaginosis flora, others normal flora, while yet others consider it to be transitional flora [39,40,41,42]. It is, therefore, hard to decide. However, as the analysis is inexpensive, the Nugent score could be considered as an alternative, particularly in places where molecular biology is not possible due to a lack of equipment or to the high cost of the equipment and reagents.

In a previous study using the same molecular tool, we observed that one-quarter of the vaginal microbiome classified as intermediate by the Nugent score could in fact correspond to bacterial vaginosis. In this new study, we observed that less than 10% of the intermediate flora could correspond to bacterial vaginosis. While molecular diagnostics has long been the preserve of specialised laboratories, the COVID-19 pandemic led to the widespread deployment and use of such equipment [43]. While various molecular diagnostic strategies have been developed, those targeting *F. vaginae* and *G. vaginalis* as markers have proved to be the most suitable [27,44]. However, molecular biology analyses remain tedious and for some costly, which may limit their use.

Other diagnostic tools for bacterial vaginosis have also been developed, such as saline microscopy, wet mount microscopy, VGTest™ ion motility spectrometry, and chromogenic tests such as OSOM^®^ BVBlue^®^, as well as an automated microbial molecular identification system, BD Affirm™ VPIII [21,45,46]. Rapid tests detecting the presence of proline amino peptidase have also shown high levels of specificity and sensitivity [45]. Finally, the home-based electrochemical rapid sensor discussed by Banks et al. [47] also represents an emerging technology. However, these techniques have not yet been and might not be adopted or integrated into routine clinical practice.

These data prompted us to reflect on the development of a new diagnostic tool that is more specific than the Amsel criteria; faster, more precise and more reproducible than the Nugent score; and less tedious and costly than molecular biology. After receiving a shared Nobel Prize in 2002, the global trend towards the use of diagnostic methods based on mass spectrometry has been apparent [48]. MALDI-TOF mass spectrometry has since been widely used as a rapid and highly analytical tool for identifying bacteria and fungi isolated in culture in the laboratory or directly from specimens such as blood cultures, cerebrospinal fluid, and urine, as well as the identification of various types of arthropods [49,50,51,52,53,54,55,56]. All of this can be achieved with low-cost reagents, with the main investment being the purchase of the MALDI-TOF device, which can also be shared between laboratories [57]. The present study used MALDI-TOF mass spectrometry as a diagnostic tool to detect bacterial vaginosis. The “VAGI-TOF” results are encouraging in terms of the diagnostic accuracy, reproducibility, speed, and cost-effectiveness. Our observations reveal a significant concordance between the molecular biology results and those obtained using the MALDI-TOF method. Only 5 out of 130 cases showed discrepancies. This strong correlation reinforces the reliability and validity of our new diagnostic tool based on MALDI-TOF for identifying bacterial vaginosis. However, several biases of the current study should be considered, such as the moderate number of samples analyzed and the fact that not all of them could be analyzed with the different diagnostic strategies.

On the whole, the diagnosis of bacterial vaginosis is not simple. Simply relying on the presence of abnormal vaginal discharge and elevated pH does not appear to be a reasonable approach. While many questions remain unanswered, such as the aetiology and possible absence of clinical manifestations with the same a priori abnormalities observed in the vaginal microbiome, the preliminary data show that MALDI-TOF mass spectrometry performed on vaginal swabs (“VAGI-TOF”) may be a future diagnostic strategy.

## Figures and Tables

**Table 1:** Main clinical characteristics of the 130 patients.

Main Characteristics	N = 130	%
*Participant’s status*		
Reproductive age without pregnancy	110	84.7%
Pregnancy	17	13%
Postmenopausal	3	2.3%
*History of bacterial vaginosis*		
Yes	34	26%
No	96	74%
*Medical contraceptive method*	*39*	*30%*
Estrogen/progesterone	17	43.6%
Progesterone only	5	12.8%
Copper intrauterine device	14	35.9%
No data	3	7.7%
*Reason for consultation*		
Abnormal vaginal discharge	62	47.7%
Gynecological follow-up	31	23.9%
Pelvic pain	16	12.3%
Others	21	16.1%

**Table 2:** Comparison of the performances of the diagnostic strategies used for the detection of bacterial vaginosis in 130 women (* patient numbers).

Discharge + vpH ^£^	Amsel-Like Criteria	Nugent Score	Molecular Biology	MALDI-TOF	Selected Diagnosis (*)	Interpretation of Results
**5 diagnostic strategies applied to 84 women**						
NF	NF	NF	NF	NF	NF (28)	Concordance of the 5 diagnostic strategies
BV	BV	BV	BV	BV	BV (14)	Concordance of the 5 diagnostic strategies
NF	NF	IF	NF	NF	NF (14)	Difficulty to classify the IF identified with NS
BV	BV	IF	BV	BV	BV (1)	Difficulty to classify the IF identified with NS
BV	NF	NF	NF	NF	NF (4)	False positive of vaginal discharge + vpH
NF	NF	BV	NF	NF	NF (2)	False positive of NS
NF	BV	BV	BV	BV	BV (3)	False negative of vaginal discharge + vpH
BV	BV	BV	BV	NF	BV (1)	False negative of MALDI-TOF
BV	NF	IF	NF	NF	NF (3)	Difficulty to classify the IF identified with NS and false positive of discharge + vpH
NF	BV	IF	NF	NF	NF (3)	Difficulty to classify the IF identified with NS and false positive of Amsel-like criteria
BV	BV	NF	NF	NF	NF (2)	False positive of vaginal discharge + vpH and Amsel-like criteria
BV	NF	NF	NF	BV	NF (1)	False positive of vaginal discharge + vpH and MALDI-TOF
NF	NF	BV	BV	NF	NF (1)	False positive of NS and molecular biology
BV	BV	BV	NF	NF	BV (1)	False negative of molecular biology and MALDI-TOF
NF	NF	BV	BV	BV	BV (1)	False negative of vaginal discharge + vpH and Amsel-like criteria
BV	BV	IF	NF	NF	unconclusive (4)	Too many discrepancies between the different strategies to conclude
BV	NF	IF	NF	BV	unconclusive (1)	
**4 diagnostic strategies applied to 33 women**						
NF	NF	Np	NF	NF	NF (17)	Concordance of the 4 diagnostic strategies
BV	BV	Np	BV	BV	BV (4)	Concordance of the 4 diagnostic strategies
NF	BV	Np	NF	NF	NF (1)	False positive of Amsel-like criteria
NF	BV	Np	BV	BV	BV (2)	False negative of vaginal discharge + vpH
BV	BV	Np	NF	BV	BV (1)	False negative of molecular biology
BV	BV	Np	NF	NF	unconclusive (9)	Too many discrepancies between the different strategies to conclude
**3 diagnostic strategies applied to 12 women**						
NF	Np	Np	NF	NF	NF (2)	Concordance of the 3 diagnostic strategies
BV	Np	Np	BV	BV	BV (3)	Concordance of the 3 diagnostic strategies
BV	Np	Np	NF	NF	NF (5)	False positive of vaginal discharge + vpH
NF	Np	Np	BV	BV	BV (1)	False negative of vaginal discharge + vpH
**2 diagnostic strategies applied to 1 woman**						
Np	Np	Np	NF	NF	NF (1)	Concordance of the 2 diagnostic strategies

^£^ Vaginal discharge + vaginal pH; NF: normal flora; BV: bacterial vaginosis; NS: Nugent score; Np: not performed. The background color indicates the discordance of the strategies used.

**Table 3:** Comparison of the performances of the bacterial vaginosis diagnostic tools (a) with the Nugent score as the reference tool and (b) with molecular biology as the reference tool.

(a)									
Diagnostic Strategies	Bacterial vaginosis (with the Nugent score * as the reference tool)								
	Sens %	95% CI	Spec %	95% CI	PPV %	95% CI	NPV %	95% CI	Accuracy %
Discharge + vpH	69.6	(47.08–86.79)	80	(63.06–91.56)	69.6	(52.77–82.38)	80	(67.84–88.35)	75.9
Amsel-like criteria	82.6	(61.22–95.05)	94.3	(80.84–99.30)	90.5	(70.94–97.37)	89.2	(77.13–95.28)	89.7
Molecular biology	87	(66.41–97.22)	100	(90–100)	100	-	92.1	(80.24–97.10)	94.8
MALDI-TOF	78.3	(56.30–92.54)	97.1	(85.08–99.93)	94.7	(72.04–99.21)	87.2	(75.76–93.67)	89.7
**(b)**									
**Diagnostic strategies**	**Bacterial vaginosis (with molecular biology as the reference tool)**								
	**Sens %**	**95% CI**	**Spec %**	**95% CI**	**PPV %**	**95% CI**	**NPV %**	**95% CI**	**Accuracy %**
Discharge + vpH	76.2	(52.83–91.78)	74.6	(62.06–84.73)	50	(38.08–61.92)	90.4	(81.19–95.34)	75
Amsel-like criteria	90.5	(69.62–98.83)	84.1	(72.74–92.12)	65.5	(51.42–77.33)	96.4	(87.59–99.00)	85.7
Nugent score ^£^	95.2	(76.18–99.18)	95.2	(86.71–99.01)	87	(68.75–95.28)	98.4	(89.85–99.75)	95.2
MALDI-TOF	90.5	(69.62–98.83)	96.8	(89.00–99.61)	90.5	(70.69–97.40)	96.8	(89.08–99.13)	95.2

* Nugent scores of 0 to 3 were considered negative and 7 to 10 positive. Women with Nugent scores between 4 and 6 (corresponding to intermediate flora) were excluded from this analysis. **^£^** Nugent scores of 0 to 6 were considered negative and 7 to 10 positive. Sens: sensitivity; Spec: specificity; PPV: positive predictive value; NPV: negative predictive value.

**Table 4:** Performance of MALDI-TOF mass spectrometry compared to the results of the combination of abnormal discharge and pH, Amsel-like criteria, Nugent score, and molecular biology.

Variables	N	Sens % (95% CI)	Spec % (95% CI)	PPV % (95% CI)	NPV % (95% CI)	Accuracy %
Discharge + vpH	129	46.3 (32.62–60.39)	90.7 (81.71–96.16)	78.1 (62.51–88.44)	70.1 (64.43–75.22)	72
Amsel-like criteria	118	54.3 (39.01–69.10)	95.8 (88.30–99.13)	89.3 (72.74–96.30)	76.7 (70.49–81.88)	79.7
Nugent score ^£^	84	78.7 (56.30–92.54)	97.1 (85.08–99.93)	94.7 (72.04–99.21)	87.2 (75.76–93.67)	89.7
Molecular biology	130	93.6 (78.58–99.21)	97 (91.40–99.21)	90.6 (75.96–96.73)	98 (92.62–99.46)	96.2

**^£^** Nugent scores of 0 to 6 were considered negative and 7 to 10 positive. Sens: sensitivity; Spec: specificity; PPV: positive predictive value; NPV: negative predictive value.

## Data Availability

Data are contained within the article and supplementary materials.

## Supplementary Materials

The following supporting information can be downloaded at: https://www.mdpi.com/article/10.3390/microorganisms12010111/s1. Table S1: Results of the five diagnostic strategies for bacterial vaginosis according to the presence or absence of abnormal vaginal discharge; (a) for all vaginal samples; (b) for the 84 vaginal samples analyzed using the five diagnostic strategies.
