# Supplementary material for: Evaluation of Various Diagnostic Strategies for Bacterial Vaginosis, Including a New Approach Based on MALDI-TOF Mass Spectrometry
Source: Microorganisms. 2024 Jan 5;12(1):111. doi: 10.3390/microorganisms12010111 (PMC10821145; doi:10.3390/microorganisms12010111)
Supplement: Supplementary file 1 [file microorganisms-12-00111-s001.zip › microorganisms-2755055-supplementary.pdf]

Supplementary Table S1. Results of the five diagnostic strategies for bacterial vaginosis according to the presence or absence of abnormal vaginal discharge; (a) for all vaginal samples; (b) for the 84 vaginal samples analysed by the five diagnostic strategies.

a.

	**Total of 130 women (Percentage)**							
	**68 with abnormal vaginal discharge (52.3%)**				**62 without abnormal vaginal discharge (47.7%)**			
	**Vaginal flora type**				**Vaginal flora type**			
**Diagnostic tools**	**NF**	**IF**	**BV**	**Np**	**NF**	**IF**	**BV**	**Np**
**Discharge + vpH**	13 **(19.1%)**	-	54 **(79.4%)**	1 (1.5%)	62 **(100%)**	-	0	-
**Amsel-like criteria**	21 **(30.9%)**	-	37 **(54.4%)**	10 (14.7%)	51 **(82.3%)**	-	9 (14.5%)	2 (3.2%)
**Nugent score**	12 **(17.6%)**	15 (22%)	16 **(23.5%)**	25 (36.8%)	23 **(37.1%)**	11 **(17.7%)**	7 (11.3%)	21 (33.9%)
**Molecular biology**	45 **(66.2%)**	-	23 **(33.8%)**	-	54 **(87.1%)**	-	8 (12.9%)	-
**MALDI-TOF**	43 **(63.2%)**	-	25 **(36.8%)**	-	55 **(88.7%)**	-	7 **(11.3%)**	-

NF: Normal flora; IF: Intermediate flora; BV: Bacterial vaginosis; Np: Not performed

b.

	**Total of 84 women (Percentage)**					
	**43 with abnormal vaginal discharge (51.2%)**			**41 without abnormal vaginal discharge (48.8%)**		
	**Vaginal flora type**			**Vaginal flora type**		
**Diagnostic tools**	**NF**	**IF**	**BV**	**NF**	**IF**	**BV**
**Discharge + vpH**	11 **(25.6%)**	-	32 **(74.4%)**	41 **(100%)**	-	**0**
**Amsel-like criteria**	20 **(46.5%)**	-	23 **(53.5%)**	35 **(85.4%)**	-	6 **(14.6%)**
**Nugent score**	12 **(27.9%)**	15 **(34.9%)**	16 **(37.2%)**	23 **(56.1%)**	11 **(26.8%)**	7 **(17.1%)**
**Molecular biology**	27 **(62.8%)**	-	16 **(37.2%)**	36 **(87.8%)**	-	5 **(12.2%)**
**MALDI-TOF**	26 **(60.5%)**	-	17 **(39.5%)**	37 **(90.2%)**	-	4 **(9.8%)**

NF: Normal flora; IF: Intermediate flora; BV: Bacterial vaginosis.
